# Supplementary material for: Are carbon emissions trading and green financial instruments synergistic? -Comprehensive quantitative research based on content analysis
Source: PLoS One. 2024 Mar 7;19(3):e0298601. doi: 10.1371/journal.pone.0298601 (PMC10919601; doi:10.1371/journal.pone.0298601)
Supplement: S1 Data — (ZIP) [file pone.0298601.s001.zip › Data/地级市Z_F工作报告.zip!/╟α║ú/╬≈─■╩╨/└┤╫╘╣1⁄2╓┌║┼í╛┬φ┐╦╩2╛▌═°í┐_2018.docx]

各位代表：

现在，我代表市人民政府向大会报告工作，请予审议，并请市政协委员和其他列席人员提出意见。

一、2017年工作回顾

2017年，是全市上下攻坚克难、聚力奋进、大有收获的一年。在省委、省政府和市委的坚强领导下，我们深入学习贯彻党的十九大精神，以习近平新时代中国特色社会主义思想为指导，坚持稳中求进工作总基调，全面贯彻新发展理念，以供给侧结构性改革为主线，按照省十三次党代会、市十四次党代会和省市全会的安排部署，牢固树立“四个意识”，忠实践行“两个绝对”，以“四个转变”推动落实“四个扎扎实实”重大要求，发扬干在实处、走在前列的精神，稳增长、促改革、调结构、惠民生、保生态、防风险等各项工作扎实推进，绿色发展样板城市和幸福西宁建设取得了新成就。

——迎难而上、激流勇进，经济发展稳中向好。深入开展“百日攻坚”等四大专项行动，出台促进投资、助推实体经济发展等系列政策措施。保持了强劲的经济发展势头，全年实现地区生产总值1284.91亿元、增长9.5%，对全省经济增长贡献率达66.2%，全社会固定资产投资1600.03亿元、增长14.3%，地方公共财政预算收入79.16亿元、同口径增长18.2%。主要指标均高于全省水平，对全省经济平稳增长支撑作用持续攀升。结构调整稳步推进，三次产业结构调整为3.3:43.3:53.4，服务业对经济增长的贡献率达到44.2%，形成二三产业协同拉动经济增长的格局。基础设施、工业、房地产投资保持稳定，民间投资增速由负转正，新兴产业投资增长45.9%，以投资促进结构优化态势明显。“青洽会”“双50”等重大招商行动成果丰硕，比亚迪等行业领军企业落户西宁，签约项目124个，到位资金909.92亿元。对外开放成果丰硕，成功举办“丝绸之路沿线国际友城峰会”，新增国际友城和友好交流城市5个，西宁机场年旅客吞吐量突破500万人次。在中亚和中东欧开展西宁城市宣传及商贸旅游推介活动，自营商品进出口总额增长29.6%。

——全盘谋划、提质增效，产业转型升级步伐加快。把培育“增长点增长极增长带”作为转型升级的着力点，全力推进“双百”工程，持续优化产业结构。农业转型发展加快，着力构建现代农业三大体系，新建蔬菜标准化、规模化生产基地2万亩，新培育农民合作社872家、家庭农牧场217家、省级以上龙头企业15家，汇丰景园田园综合体建成运营，农业总产值连续13年增长5%以上。新旧动能转换加速，千亿锂电基地、光伏制造中心等绿色产业发展势头强劲，三江源生态与高原农牧业和政企共建的国家光伏等重点实验室落户西宁，中藏药产业集群等列入国家创新型产业集群试点，新增高新技术企业12家，高新技术和新能源产业增加值分别增长22.8%、40.8%。实施市级以上科技项目148项，申请专利2442件，授权1187件。顺利通过国家创新型城市试点验收。关停大通煤矿120万吨落后产能，粗铅、水泥产量分别下降77.2%、27.4%，商品住房库存面积下降112.6万平方米。落实减税降费政策，累计降低企业各类成本17.2亿元。现代服务业蓬勃发展，成功举办第二届中国西部自驾游联盟年会和西部自驾游产业发展论坛，成立西宁文旅发展公司，全年共接待国内外游客2138.29万人次，实现旅游收入250.97亿元，分别增长15.4%、28.2%；西宁金融控股集团、西宁信用担保集团挂牌运行，金融业增加值增长8%。顺利入选国家电子商务示范城市，“阿里巴巴·西宁产业带”上线运营，电商交易额350亿元、增长20%。新增市场主体2.67万户、中小微企业8176户。

——纵横布局、精准发力，城市转型升级成效显著。坚持提高标准，拓展城市空间，提升城市品位，努力补齐发展短板。城市承载能力明显增强，围绕“一芯双城、环状组团发展”的生态山水城市格局，着力构建绿色发展空间体系，编制完成多巴新城总规、南川文化旅游商贸会展区城市设计及风貌导则，提升了职能定位明确了发展方向。高标准推进多巴新城建设，新华联国际旅游城、青海丝绸之路国际物流城全面实施；南川片区市政设施、市民中心等引导性项目开工。“畅通西宁”开工道路101条，昆仑桥、湟水路高架等53项工程建成通车，人均城市道路面积由8.3平方米提高到9.5平方米；推进公交都市建设，建成首条公交专用道，完成掌上公交等智能交通系统建设；整治改造停车场泊位1.5万个，建成绿道400公里。拥堵指数从3.7下降至2.5，缓堵保畅初见成效。城乡环境持续优化，启动“城市双修”试点，苦水沟景观治理、香水泉遗址恢复重建工程等项目进展顺利。海绵城市开工试点项目130项。累计建成综合管廊26公里，建设理念和工艺水平走在全国前列。深化“美丽夏都·清洁西宁”品牌建设，121条主次干道全时段机械化洗扫率达到80%，在415个小区（单位）实行生活垃圾分类试点。改建农村公路268公里。提高了14.4万人饮水安全保障水平。启动4条乡村旅游示范带，实施1镇21村风貌提升工程，建成美丽乡村77个，完成100个村环境整治试点项目。

——绿色为先、多措并举，生态底板更加厚实。扎实开展“高原绿、西宁蓝、河湖清”建设行动，湟源县获评全国生态文明建设示范县。国土绿化规模空前，三北、西堡生态森林公园等林业生态工程造林36万亩，南北山完成高标准造林7万亩，28年绿化成果得到国家肯定。园博园完成总规编制和绿化整地。新改建游园、街头绿地14处、道路绿地25条，提升绿道景观75.8公里，新增城区绿地面积1147.5亩。大气质量持续改善，严格落实五个“100%”，GPS定位管理渣土车，淘汰黄标车4324辆，“煤改气”191.5蒸吨。出台新能源汽车推广应用方案，新建充电桩1034个，新增纯电动公交车55辆。全年空气优良率达到81.1%，空气质量指数和排名位居西北省会城市第一。水污染治理扎实有效，建立完善四级复合型河长组织体系，实现河长管理全覆盖。全国水生态文明城市建设试点通过验收。关停、搬迁禁养区规模化畜禽养殖场66家，整治湟水河干支流排污口75个，8个国省控断面水质全部达标，国控出境小峡桥断面消除劣V类水体。以中央环保督察为契机，强力推进环保问题整改，规范整治各类污染企业838家，解决污染问题1000余个，一大批群众关心的环保问题得到有效解决。

——以人为本、紧贴民心，民生保障全面提升。坚持80%以上的财力用于改善民生，精准扶贫、畅通西宁、环境保护等十大类40件民生实事全面完成！公共服务能力不断增强，健康西宁全面推进，实施医疗集团医保打包付费方式改革，探索家庭医生“3+1+N”团队、健康签约“承包”服务新模式，重点人群签约率达75.2%。成立12个城乡义务教育集团，组建现代服务业职教集团，三县顺利通过国家义务教育发展基本均衡县评估认定，建立了覆盖学前到高等教育学段的资助体系，惠及学生46.3万人次。新改建基层综合文化服务中心236个。城区10分钟健身圈范围逐步扩大，全市行政村健身设施实现全覆盖。全运会以1金1铜成绩实现竞技体育奖牌零的突破。全面完成年度脱贫任务，严格落实“八个一批”精准脱贫措施，加大行业扶贫和东西部扶贫协作力度，整合各类涉农资金25.46亿元，实现100个贫困村退出。兜底保障标准显著提升，城市最低生活保障金每月提高50元，农村最低生活保障金每年提高350元。新增城镇就业3.18万人，农村劳动力转移就业39.81万人次。投入2.6亿元保障城镇困难和低收入家庭基本生活。150个农村老年之家投入使用，为6.8万名困难和高龄老人购买养老服务。改造棚户区1.87万套、农村危旧房6500户。新建蔬菜平价店71家，居民消费价格指数控制在1.8%。城市安全发展呈现良好局面，国家食品安全示范城市创建工作稳步推进。全市连续16个月未发生较大及以上生产安全事故。实施社会治安分级评价机制改革，社会治安“八类案件”七年连降。

各位代表，在经济下行压力增大、形势异常复杂多变的情况下，市委不断强化党对经济工作的领导，针对新情况新问题，及时加强经济走势研判掌控，创造性地把中央和省委一系列决策部署落实到西宁工作实践中，确保了全市经济沿着正确方向发展。一年来，市政府全面贯彻市委决策部署，紧紧围绕脱贫攻坚、结构调整、畅通西宁、环保督察、文明创建聚力攻坚，实现了重点工作的新突破。

一年来，我们加速转型、实干引领，绿色发展的实践取得新突破。出台建设绿色发展样板城市实施意见，成立绿色发展委员会，实施六大建设行动，西堡生态森林公园、园博园等重大生态修复工程启动，新能源新材料等绿色产业势头迅猛，成功举办绿色发展样板城市论坛，湟水河更加清澈透明，城市园林由绿到美，大气和水环境质量实现双提升。

一年来，我们一心为民、矢志不渝，幸福西宁的实惠更加丰盈。2.39万农村贫困人口稳定脱贫，3.9万城镇困难群体生活得到保障，提高了养老、低保、医疗保障标准。城镇居民收入跨上3万元台阶，农村居民收入突破万元，“畅通西宁”拉伸城市骨架，群众出行更加便捷。“清洁西宁”改善环境，品牌建设广受赞誉。在中国社科院组织的生活质量主观满意度第三方测评中排名第一。

一年来，我们凝心聚力、锲而不舍，文明创建的多年追求结出硕果。自觉履行守护民族团结生命线的政治责任，成功创建全国民族团结进步示范市，书写了民族团结进步工作的生动实践。将人民对美好生活的向往作为奋斗目标，践行“为谁辛苦为谁甜”的创建宗旨，省市共建，全民上阵，历经八年接续奋斗，赢得全国文明城市这块“金字招牌”，市民自豪感、归属感空前高涨，全市人民孜孜以求的梦想终于成真！

一年来，我们坚持问题导向、彰显特色，重点改革走在了前列。公立医院改革和四级紧密型医联体改革受到国务院通报表扬。湟源县农村土地制度三项改革试点取得实质性进展。“放管服”改革持续深化，率先构建了“一目录六清单”政府监管服务新模式，网上审批率达到85%。法治政府建设位居西北省会城市第一。一批具有标志性、关联性作用的重大改革顺利推进。

各位代表，过去的一年，我们担当有为，切实转变政府职能，不断强化服务功能，政府效能不断提升。严格落实全面从严治党要求，自觉执行党风廉政建设责任制，干部作风明显好转。坚决落实市委各项工作部署，实行重大问题集体研究、重大事项向市委报告制度。认真执行人大及其常委会的决议、决定，主动接受人大工作监督、法律监督，自觉接受政协民主监督，办理答复人大代表建议176件、政协委员提案190件，办结率100%。广泛听取民主党派、工商联、无党派人士意见建议，国防动员、双拥、普法、民族宗教、新闻广电出版、外事侨务、人防、气象、地震、供销合作、工青妇、慈善、残疾人、红十字等工作全面健康发展。

各位代表，回顾2017年，压力挑战超乎预期，答卷成绩令人欣慰。这是省委省政府亲切关怀的结果，是市委坚强领导的结果，是市人大、政协大力支持的结果，是全市人民同心协力、拼搏奋斗的结果。在此，我代表市人民政府，向全市各族干部群众，向人大代表、政协委员、各民主党派、工商联、无党派人士和各人民团体，向驻宁部队指战员、武警官兵、公安干警和驻宁企业，向所有关心、支持西宁建设发展的同志们、朋友们，致以崇高的敬意和衷心的感谢！

我们也清醒地认识到，西宁的发展还存在一系列不平衡不充分的问题，主要表现为：发展质量效益不优，“稳”的基础不牢固；新旧动能转换尚需时日，民营经济发展不充分；城市规划建设标准还不够高，管控不全面；开放水平与先进城市和自身定位还有差距，国际化程度有待提高；民生社会事业发展不均衡，社会治理创新亟待加强；部分政府工作人员综合素质不能适应新要求，“四风”问题仍然存在。我们一定正视问题，切实推进解决。

二、2018年主要目标和重点任务

根据中共西宁市委十四届六次全会的部署，今年政府工作的总体要求是：坚持以习近平新时代中国特色社会主义思想为指导，全面贯彻党的十九大和十九届一中、二中全会及十九届中央纪委二次全会、中央经济工作会议、中央农村工作会议精神，牢固树立“四个意识”，不断坚定“四个自信”，始终做到“三个一以贯之”和“五个过硬”，忠实践行“两个绝对”，坚持和加强党的全面领导，牢牢把握稳中求进工作总基调，自觉贯彻新发展理念，紧扣我国社会主要矛盾变化在西宁的具体表现，统筹推进“五位一体”总体布局和协调推进“四个全面”战略布局，全面落实省第十三次党代会、省委十三届三次全会和市第十四次党代会以及市委十四届五次、六次全会部署，围绕以“四个转变”落实“四个扎扎实实”和“三高一新”的要求，坚持生态优先、发展率先、精神领先，坚持以供给侧结构性改革为主线，统筹推进稳增长、促改革、调结构、惠民生、防风险各项工作，聚力建设现代产业体系、推动高质量发展，聚力推进改革开放、依法治市，聚力打好防范化解重大风险、精准脱贫、污染防治三大攻坚战，聚力实施乡村振兴战略、区域协调发展战略，聚力推进党的建设新的伟大工程，在新的起点上加快打造绿色发展样板城市，奋力开创新时代建设幸福西宁新局面。

主要预期目标是：地区生产总值增长9%左右；固定资产投资增长8%；社会消费品零售总额增长9%；地方公共财政预算收入增长7.5%；全体居民人均可支配收入增长9%；城镇新增就业3万人，城镇登记失业率控制在2.5%以内；城市空气质量优良率、居民消费价格指数、节能减排完成省定目标。

以上目标，主要是按照新时代的新要求，突出稳中求进、高质量发展，既考虑自身发展条件和可行性，也考虑支撑全省经济增长和正向引导预期，并与“十三五”规划目标相衔接，经过全市上下共同努力是可以完成的。

围绕以上目标，我们将着力在七个方面展现新作为。

（一）全力推进绿色产业建设行动，在推动高质量发展上要有新作为。把握建设现代化经济体系的目标和重点，培育点极带经济增长新格局，加快新旧动能转换，着力构建现代产业体系，推动高质量发展。

加速培育新兴产业。实施新兴产业倍增计划，注重清洁能源与绿色产品联动，力争高技术产业产值年均增长20%以上，“十三五”末实现翻番，占比提高至20%以上。锂电抢占储能产业制高点，依托比亚迪、泰丰先行等龙头企业，以动力及储能电芯制造为核心，大力发展锂电配套产业，加速打造全产业链条，电芯产能达到8.5吉瓦，夯实千亿锂电基地基础。光伏光热以市场换投资，推动光伏电站建设开发与上游光伏制造业联动发展，依托亚洲硅业、国电投等骨干企业，加快构建应用与制造垂直一体化发展新模式，晶硅产能达到3万吨，组件产能达到1吉瓦。生物医药激活闲置药号，依托和引进一批龙头企业，实施兼并重组和技改扩能，推动重大疾病治疗药物创新和产业化，产值增长15%以上。

改造提升传统产业。探索推进网络化协同制造、个性化定制、服务型生产新模式，实施一批智能工厂、数字化车间试点项目，推动传统制造业向数字化、网络化、智能化、绿色化转变。铝镁合金瞄准汽车轻量化等发展方向，依托绿色电、盐湖镁、西宁铝产业基础，建设北川绿色铝镁合金高新材料产业园，加快园区基础设施建设，引进变性镁合金等一批标志性项目。农牧产品精深加工利用湟源背靠大牧场、面向大市场的地缘优势和集散功能，发挥“无公害超净区”外溢效应，推进青藏高原原产地特色产业聚集园建设，打造高原绿色有机品牌的高地，完成园区规划，开展园区建设。藏毯绒纺利用“互联网+”等新技术新模式拓宽市场，再开办一批境外营销网点和分拨营销中心，提升“藏毯之都”品牌力。装备制造逐步向数控化、智能化迈进，加快升华电梯智能制造、大美煤业智能工厂等重点项目。

积极推进创新驱动。对接“中国制造2025青海行动方案”，以“互联网+先进制造业”为重点，实施高新技术和科技型企业“双倍增”“科技小巨人”培育计划。建成西宁科技大市场，理顺青海国家高新技术产业开发区体制机制，建成铝镁合金研发中心等3个重点产业技术创新平台，全年专利申请量、授权量、每万人有效发明专利拥有量均增长20%。实施专利奖励资助政策，探索开展专利权、知识产权质押融资试点。落实“质量强国”战略，聚集创新人才，培育企业家精神，弘扬劳模和工匠精神，实施“引才聚才555计划”。

发挥园区引领作用。持续巩固现代产业集群向园区聚集发展的良好态势，落实好重点项目服务联系制度和派驻联络员制度，用好产业基金，引导更多资金投向战略性新兴产业。支持开发区引领示范全市绿色低碳循环发展，推动甘河、东川、南川园区循环化改造和甘河园区低碳示范试点取得实效，规上工业单位增加值能耗下降2%以上。实施智慧园区建设工程，加快申报国家级产城融合示范区。三县要依托工业、农业、旅游等产业园，大力发展非公经济，吸引更多企业入驻，实现错位发展，加速提升县域经济实力。

（二）全力推进治理能力建设行动，在城市高品位建设上要有新作为。把握城市群发展规律，增强规划引领作用，提高规划建设品位，推动扩容提质，建设一流城市。

规划引领扩容提质。深度融入兰西城市群建设。深刻把握新时代进入以城市群为主体，大中小城市协同发展的城市发展规律，积极融入兰西城市群战略。做好规划衔接，主动承担核心城市职能，提升“一带一路”中的区域地位。促进重点基础设施互联互通，强化兰西主轴，构建开放合作的城市群主骨架。推进城市群重大产业布局重塑，加强沿线城市产业分工协作。更加突出西宁辐射服务藏新、连接川滇的战略支点功能，打造具有一定国际影响力的现代化中心城市。促进以西宁为中心的东部城市群一体化发展。围绕建设西宁周边1小时产业圈、物流圈、旅游圈、生态圈、交通圈，推进G6高速北移等“三环七射”线网项目建设，发挥核心引领作用，促进西宁从首位度高向影响大、辐射远、带动强转变，形成东部城市群大中小城市网格化格局。着力推动城市空间重构。围绕“一芯双城、环状组团发展”生态山水城市格局，加快建设园博园和西堡生态森林公园，早日建成城市“绿芯”，勾画生态山水城市的点睛之笔。着力推动“双城联动”，有序推进湟中撤县设区，实施多巴湖、扎麻隆湿地等生态本底工程，推进新华联国际旅游城等标志性项目，按照绿色开放和生态宜居现代化一流城区的高标准，打造多巴城市副中心，加快疏解中心城区超负荷公共服务和居住职能，增强、拓展服务全省的功能和空间。围绕建设高品质城市形象展示区，完善南川基础设施，加快建设市民中心，启动建设国际会展中心，打造融入“一带一路”对外开放新平台和引领全省高端服务业的增长极。

提高标准严格管控，打造一流城市。提升规划理念。围绕高标准建设绿色发展样板城市，按照后现代城市规划建设新理念新理论，以“城市让生活更美好”为价值追求，以构建高原生态山水城市为目标，把以人为本、尊重自然、传承历史、绿色低碳等理念融入城市规划全过程。提升全域规划编制水平，以主体功能区规划统筹市域各类空间规划，推进“多规合一”，形成市域规划一张图。追求高品位建设。以“治山、理水、润城”海绵城市原则，为干燥的西宁多留一份湿润，新建项目实现海绵城市要求全覆盖，已建成区逐步实施海绵化改造，全面完成海绵城市示范区试点项目。将火烧沟、苦水沟等城市生态修复和水井巷、卫城东北片区等城市修补项目打造成“城市双修”的示范。制定综合管廊建设管理办法，新建道路与综合管廊一体设计、同步建设，已建成区逐步推进管廊建设，完成46公里建设任务。严格执行居住小区配建学校、托幼、医疗、交通、绿化等规划要求。新建建筑全面执行绿色建筑标准，加快发展装配式建筑，鼓励使用高品质建筑外立面装饰材料和新型绿色建材。推进“畅通西宁”绿色交通升级版，新开工道路18条，建成凤凰山路等38条道路，基本完成“畅通西宁”三年行动计划，人均道路面积达到12.3平方米。加快地下通道、人行天桥等便民设施建设；继续实施公交专用道建设，全面建成“公交都市”；完善智能停车诱导系统，全面推行公共停车场电子收费；新增绿道65公里，构建畅通便捷的绿道网络；在党政机关和出租、公交、市政、配送领域优先推广新能源汽车，再新增充电桩1000个。严格规划管控。持续推进“双增双减”，严格限定住宅项目容积率上限，严格控制中心城区高层住宅建筑和沿街住宅小区底商建设，住宅建筑与商业建筑单独设置；充分利用城市拆迁腾退地和边角地、闲置地开展绿化建设。严查严管违法建设、违法用地，持续开展拆违打非行动。注重主城区留白，加强城市修补，避免大拆大建，强化重点地段城市设计，严格管控中心广场、火车站、南北山等重要节点的特色风貌，对昆仑大道、海南路、机场高速、湟水河等城市主轴线、视线通廊、主干道、河道建筑严格红线退界、建筑高度、连续长度以及建筑形态控制，协调景观风貌，实现轴线、天际线、文脉、风格和色彩的协调统一。三县落实海绵城市、绿色建筑等要求，引入城市设计，推进控规全覆盖，确保重点城镇总规全覆盖、村庄规划全覆盖，形成全域空间布局、空间管制、生态保护、城镇布局、基础设施一体化的规划管控和规划引导体系。

推进城市管理再精细，营造整洁有序环境。打造“美丽夏都·清洁西宁”升级版，坚持“随手拍”“航拍巡查”等成功做法，推动机械化作业向背街小巷延伸，扩大道路清洗范围，健全街长制等长效机制，严格“门前三包”责任制，加强国省道、景区、农贸市场等重点区域环卫管理，推动“清洁西宁”向农村延伸。深入推进城管体制改革，划转市政环保等相关领域部分行政处罚权，继续推动执法重心和执法力量向基层下移下沉，发挥街道综合执法指挥中心主体作用，全面落实属地监管责任。实施厕所革命三年行动计划。推进生活垃圾分类，启动垃圾焚烧发电项目，全面提升生活垃圾规范化处理水平。

（三）以“高原绿”“西宁蓝”“河湖清”建设行动为抓手，在生态文明建设上要有新作为。环境就是民生、青山就是美丽、蓝天也是幸福，用心呵护西宁好山好水好风光。今年，市委市政府将切实加大绿色投入，统筹各类资金10亿元推进绿色发展。

以“高原绿”建设行动实现由绿到美转变。弘扬尕布龙两山绿化精神，实施国土绿化提速工程，按照三年造林156万亩规划，今年造林绿化51.5万亩。抓好西堡生态森林公园火烧沟综合治理、湟中水滩花海景区建设，完成造林3.3万亩、作业道路150公里；完成园博园1800亩防护林区域绿化及道路、灌溉系统等部分土建工程。南北山三期造林10.1万亩，提前两年完成造林目标。启动湟水流域国家百万亩规模化林场试点建设，筹备好全国造林绿化观摩现场会。实施城区园林增绿提质工程，利用环保督察和“畅通西宁”拆迁空地，启动5000亩绿地建设，新建湟岸巷等10个精品主题公园游园，新改建街头绿地20处，新建改造黄河路等8条道路绿化景观，新增园林绿地面积1200亩。全面提升养护水平，引进专业化的生态修复和管护队伍，对各类绿地分等级进行养护管理。

以“西宁蓝”建设行动持续改善大气质量。制定蓝天保卫战三年行动计划，迎接国家“气十条”考核，编制大气环境限期达标规划，空气质量综合指数和排名持续走在西北省会城市前列。开展大气污染源解析，实施网格精细化监管监测，实现精准治污。落实智慧化建筑工地监管措施，进一步降低建筑施工扬尘污染。强化机动车尾气治理，对黄标车、渣土车进行全天候无缝隙监管。加大“煤改气”治理，天然气管网覆盖区域燃煤锅炉全面清零。加强餐饮油烟治理。实施重点行业污染治理专项行动，对所有重点工业污染源实行24小时在线监控。推进区域大气污染防治协作，积极有效应对防控重污染天气。

以“河湖清”建设行动打造湟水河生态活力轴。以湟水河生态廊道为主轴，南北向生态走廊为拓展，大型滨水公园为绿色开放核心，打造拥水发展的城市主轴线。抓好湟水流域水环境综合治理与可持续发展试点，推进海湖湿地公园、湟水河生态综合治理和元堡子湿地建设，建成北川滨水中心及滨水公共活动带，启动南川河生态保护项目。开工湟岸公园、锦绣公园、提升改造人民公园景观，建设三河交汇城市客厅。制定湟水河流域沟道治理方案，两年内完成剩余沟道治理。抓好“河湖库渠连通”工程，连通西干渠、国寺营渠和解放渠，促进工程治水向生态治水、系统治水转变。加快推进涉水体制改革，完善四级复合型河长体系，完成“一河一档”基础数据采集及河流健康评估等工作。

全面完成环保督察问题整改。紧盯环保督察12项问题，建立健全环保问题整改长效机制，搭建综合性生态环保投诉举报平台。对完成整改的进行“回头看”，做好巩固提升工作；未完成整改的采取过硬措施，确保如期全面完成。强化重点流域区域水污染和农业面源污染防治，开展土壤污染详查，分类治理；持续整治湟水河排污口，制定流域入河口设置布局规划，完成六污建设和四污扩能，加快中水回用和配套管网建设，力争出境断面水质达到IV类标准，中水回用率达到30%；强力整治工业污染，严控高耗能、高排放行业，加快黄河鑫业等重点企业除尘脱硫脱硝设施升级改造。坚持零容忍、全覆盖，持续加大执法力度，按照“谁污染、谁治理，谁损害、谁赔偿”的原则，依法严查环保违法行为。

（四）把乡村振兴作为提升幸福西宁内涵品质的重要举措，在城乡融合发展上要有新作为。乡村振兴战略是新时代“三农”工作的总抓手，按照“二十字”的总要求，注重当前和长远相结合统筹协调推进。今年，我们将设立1亿元乡村振兴战略专项资金，推动乡村振兴走在全省前列。

建立健全城乡融合发展体系。加强乡村振兴顶层设计，抓紧编制乡村振兴战略规划，坚持工业农业一起抓、城市农村一起抓，让乡村振兴战略成为城乡群众共享幸福西宁的美好事业。推动城乡基础设施一体化，制定“四好农村路”三年攻坚行动方案，新改建乡村公路160公里，推进城乡公交一体化；建设改造农村电网715公里；完成保障8.86万人的农村饮水安全巩固提升工程。加快促进基本公共服务均等化，依托集团化办学推动城乡义务教育一体化；以医疗集团建设为抓手，推动优质医疗资源向基层下沉，实现城乡医疗医保一体化。

巩固完善基本经营制度，实现共同富裕。巩固湟源农村土地制度改革成果，深化湟中、大通农村集体产权改革及资产股份权能改革试点，牢牢把准“增强村集体经济实力和活力”的改革方向，实施村集体经济“破零”工程，探索发展壮大村集体经济和创新乡村治理的新途径。加强乡镇村金融服务体系建设，加快农村“六类”产权确权发证，发挥互助资金协会作用，大力推进双基联动和“金穗服务惠万家”，发挥市信用担保集团作用，解决农民贷款难、贷款贵问题。

紧盯农业供给侧结构性改革，实现质量兴农。推进农业由增产导向转向提质导向，走高原绿色有机品牌之路。提升大通国家级现代农业示范区和湟源畜牧业绿色发展示范县创建成果，推进湟中县国家级现代农业示范区创建，建设西纳川、双新公路沿线、申中、总寨及三县生态牧场等现代农牧业产业园、科技园和创业园，增加中藏药、蔬菜等作物种植比例和附加值，提高农副产品加工转化率，延长产业链，促进农村一二三产融合发展。全面落实粮食安全行政首长责任制，稳定粮食生产。加强农业技术服务体系建设，健全乡镇农技推广机构，充实农技推广力量，推动科技兴农。加快培育种养大户、家庭农牧场、农民合作社、龙头企业、社会化组织等新型农业经营主体，鼓励扶持生产性服务业和联合社发展，增强农民合作社引领力，引导农户与现代农业有机衔接。

坚持人与自然和谐共生，实现乡村绿色发展。严格执行乡村建筑风貌导则，实施旅游干道沿线91个乡村风貌提升工程。再建75个高原美丽乡村及5个美丽城镇。开工建设5个乡镇生活垃圾填埋场和2个建制镇污水处理设施，2020年全面实现城镇生活污水和乡镇村垃圾全收集、全处理，农村无害化卫生厕所全覆盖，推进城乡环保一体化。借助绿水青山好环境开发农业生态旅游，实施10条乡村旅游示范带建设，发展精品民宿、文化体验等业态，形成乡村发展与特色产业良性互动。

打好精准扶贫攻坚战，实现脱贫目标。落实好“1+8+12”精准扶贫政策，整合各类涉农扶贫资金，扶持贫困地区发展特色产业，实现脱贫由“输血”到“造血”转变。聚焦、瞄准特定贫困群众精准帮扶，重点关注因病、因残、因学致贫及孤寡老人等特殊贫困人口脱贫，落实好脱贫巩固后续政策。积极开展东西部扶贫协作，深入推动“百企帮百村，百企联百户”精准帮扶和结对帮扶。坚持扶贫和扶志、扶智结合，激发贫困群众脱贫致富内生动力，完成150个贫困村退出、1.5万贫困人口年度脱贫目标，为决战决胜奠定决定性基础，确保如期实现6.6万贫困人口全面稳定脱贫、330个贫困村全部退出、三县达到“摘帽”标准。

传承发展提升农耕文明，创新乡村治理体系。挖掘传承西宁特色的农耕特质、民族特色、区域特点的物质文化及非物质文化遗产，深入实施文化惠民工程，扩大乡村文化覆盖，提升乡村文化服务能力，实现乡村文化兴盛。强化“三基”建设，完善村务监督、村务公开、民主议事机制，加强“三资”管理，发挥乡贤参事会作用，实现乡村善治。

（五）把提高基本公共服务能力作为提升幸福西宁内涵品质的重要途径，在保障改善民生上要有新作为。始终坚持兜底线、保基本、提品质，让市民获得感更充实、幸福感更真实、安全感更踏实！

办好人民满意的教育。发展普惠性学前教育，实施西宁市第三期学前教育行动，增加公办幼儿教育资源，新改建21个农村走（幼）教点；扩大政府购买服务力度，支持普惠性民办园发展，力争全市普惠性幼儿园比例达到80%，逐步解决婴幼儿照护和儿童早期教育服务问题，引领全省学前教育发展。全力承接省内藏区、东部地区等外来人口义务教育阶段入学需求，增强基础教育服务保障能力。优化教育资源配置，继续实施西宁八中、南大街学校等9个教育布局调整和光华中学、南川西路小学等11个消除义务教育大班额项目，完成西堡葛家学校、宝库乡中心学校等19个改薄项目，深入推进城乡义务教育集团化改革，提升义务教育均衡发展水平，缓解入学压力。坚持把立德树人作为根本任务，构建学段衔接、常态开展的德育工作体系。加快教育信息化建设，落实全面深化新时代教师队伍建设改革意见，搭建市级教育公共服务平台，扩大优质教育资源覆盖面，着力解决“择校热”“大班额”等突出问题。优化职业教育布局结构，推进多巴职教中心、湟源职校新校区等项目建设；围绕市场需求，打造品牌专业，深化校企合作、产教融合，推进中高职院校专业品牌建设，解决高质量发展下劳动力素质和结构调整不相适应的问题。

促进文化体育繁荣发展。创作推出《绿水青山·幸福西宁》等一批反映绿色发展理念的文艺作品。加快创建5A级博物馆群，加大对特色博物馆、非国有博物馆的扶持力度，建设宝玉陈等10个小型专业民间博物馆。启动沈那遗址考古发掘和遗址公园建设，开展孙家寨文物普查勘探。制定文庙、花园南街古城墙等保护方案。大力发展体育+旅游康养产业，加大体育场馆免费低收费开放力度，广泛推动全民健身运动，打造10分钟健身圈升级版，以全民健身推动全民健康。

打造全省医疗和康养服务基地。落实《健康西宁行动计划》，推进“3+1+N”全科医师团队建设，加强基层医护人员专业培训，健全家庭医生签约服务制度，精准扶贫建档立卡户签约率达到100%。深化四级紧密型一体化医联体改革，统筹推进分级诊疗、医院管理、支付方式、药品保障、监管考核等各项改革。提升医疗卫生服务能力，抓紧市妇幼保健计生服务中心、全科医生培养基地等项目建设。积极应对人口老龄化趋势，推动养老服务体系建设，开展居家和社区养老改革试点，鼓励社会力量参与养老服务。建设10个社区老年日间照料中心和50个农村老年之家，打造具有西宁特色的城市养老呼叫服务圈和农村养老互助服务圈。深入推进国家级医养结合试点城市建设，实施市中医院康养示范中心等项目，着力打造医养结合、安宁疗护、康复理疗等为一体的综合医养示范基地。

全力推进就业创业。深入实施高校毕业生就业促进和创业引领计划，高校毕业生登记就业率不低于87%。完善分级孵化联动机制，加快推动创业创新中小微企业发展。推行“企业订单、劳动者选单、培训机构列单、政府买单”的培训模式，实现技能就业和素质就业，农村劳动力转移就业35万人次。扩大社会保险覆盖范围。承接国家和省上相关政策，完善城镇职工和城乡居民基本养老保险制度。制定医疗保险跨省异地就医结算的配套措施，巩固完善医保异地就医直接结算成果。强化社会救助。推进“9+1”的社会救助体系建设，完善临时救助制度，实施重特大疾病医疗救助，统筹城乡特困人员救助供养制度。做好城镇贫困和困难群体帮扶工作，全程资助贫困家庭大学生1800名。落实困境儿童、重度残疾人及高龄补贴制度，做好农村留守儿童、妇女、老人关爱服务工作。持续提高城乡低保标准，年度增幅不低于10%。多措并举稳控物价。建立多主体供应、多渠道保障、租购并举的住房制度。发展住房租赁市场，支持专业化、机构化住房租赁企业发展。深化住房公积金改革，提升“互联网+公积金”服务水平。实施棚户区改造12700套，分配公共租赁住房2000套。加快老旧小区改造，开展加装电梯项目试点。启动“强化物业服务、建设幸福西宁”三年行动计划，提升基础服务水平。

始终坚持精神领先。以成功创建全国文明城市和全国民族团结进步示范市为新起点，让创建工作的精神成果持续发力。积极培育和践行社会主义核心价值观，弘扬时代新风，加强社会公德、职业道德、家庭美德、个人品德教育，培育道德沃土，形成向上向善、健康和谐的文明风尚，广泛开展文明村镇、文明家庭、五星级文明户创建活动，推动精神文明建设向更高水平迈进，当好全省精神文明建设的排头兵。强化城市民族工作，促进各民族交往交流交融，积极引导宗教与社会主义社会相适应，团结各族群众坚定不移跟党走中国特色社会主义道路。

推动城市安全发展。加强社会治理，深化平安西宁建设，开展扫黑除恶专项斗争，强化社会治安防控体系建设。加快安全生产领域改革发展，开展安全生产隐患排查治理和各类专项整治，坚守安全发展底线。深化国家级食品安全示范城市创建，开展“阳光厨房”行动，全面推行餐饮服务食品安全量化分级管理，年内对市区80%以上餐饮店卫生等级状况实行店外挂牌公示，探索网络订餐监管和阳光厨房网络直播联动新模式，保障市民“舌尖上的安全”。

深入开展双拥共建，促进军民融合发展，关心支持工会、共青团、工商联、红十字会、妇女、老龄、儿童、残疾人工作，积极推进仲裁、防震减灾、人防等事业发展。

各位代表，今年，我们将围绕社会保障、教育文化、医疗卫生等方面，精心谋划实施十大类35件民生实事，切实把该办的事竭力办好，做出的承诺说到做到，真正把民生实事办成顺应民意、贴近民情、排解民忧的民心工程！

（六）扩大消费促进有效投资，在夯实绿色发展和幸福西宁的基础上要有新作为。今年，财政支出的重点是，围绕绿色发展、三大攻坚战、乡村振兴、供给侧改革、民生事业等领域抓重点、补短板、强弱项，带动有效投资，释放内需潜力。

全力以赴扩大有效投资。继续发挥投资的关键作用，把好项目绿色准入关，挖潜力增后劲，不断提高投资效益。优化投资结构。紧盯绿色产业增后劲，构建现代产业体系，加大对光伏、锂电、生物医药等新兴产业投入，大幅提升新兴投资比重。紧盯城市绿色空间体系强基础，加大城市路网、供排水等基础设施建设；增加生态环保、污染治理、现代农业等领域投入，夯实绿色发展基础。紧盯幸福西宁建设补短板，加大教育、医疗、卫生、住房、扶贫等领域的投资力度，高质量满足民生诉求。加大三县资金投入，谋划推进一批特色产业、特色小镇和农村水利及电网等项目，带动县域经济发展。抓实项目工作。深入开展“项目生成年”活动，围绕推进供给侧结构性改革、兰西城市群建设、绿色发展样板城市六大建设行动，启动“十三五”中后期重大项目规划，谋划储备一批起点高、规模大、带动作用明显的重大工程。主动对接国家发展战略，围绕绿色产业发展、创新驱动、生态环保、民生事业等重点领域，全年争取专项资金增长10%以上。积极招商引资。突出战略招商、精准招商，充分利用行业协会、行业中介、行业龙头及专业研究机构等平台，紧盯战略新兴产业领军企业，开展集群式、猎头式、产业链式招商，全年招商引资到位资金880亿元。促进民间投资。落实细化鼓励民间投资政策措施，进一步放宽基础设施等领域准入门槛，千方百计激发民间投资活力。

顺应消费升级新趋势。统筹安排1亿元对外贸易和服务业发展资金。发挥消费的基础作用，发展新产品、新服务和新模式，推进实施“十大扩消费行动”，着力满足广大群众多样化、个性化消费需求，服务业增加值占经济比重达到50%以上。扩大服务消费。加快现代服务业优化升级，积极发展网络团购、体验式消费等生活性服务业，大力发展生产性服务业，培育发展一批技术改造和装备设备维修养护企业，为制造业企业提供服务。扩大信息消费。加快城乡宽带信息基础设施建设，培育发展大数据云计算、现代物流等新兴业态，完成智慧城市基础平台建设。大力发展农村电商，再建成80个农村社区电商服务站点，全年电商交易额430亿元以上。扩大旅游消费。围绕建设“一地一城一中心”，全面提升优质旅游服务，开展旅游信用体系建设行动。大力发展乡村旅游，推进全域全季旅游发展。加快建设自驾车营地、熊猫馆、南川温泉康养等项目，持续打造中国西部自驾车旅游联盟品牌，举办自驾游产业发展论坛，加快构建自驾游服务体系，加速西部自驾游产业聚集。全年旅游人数、收入分别增长13%和20%。

（七）深化改革扩大开放，在激发绿色发展和幸福西宁活力动力上要有新作为。以改革构建推动绿色发展的体制机制，以开放推动经济高质量发展，着力破解实体经济发展的难点问题。

深化供给侧结构性改革。推动资源要素向实体经济集聚、政策措施向实体经济倾斜、工作力量向实体经济加强，营造脚踏实地、勤劳创业、实业致富的发展环境和社会氛围，打造全省改革高地。落实“去降补”任务，推动铁合金等两高一低行业化解过剩产能，关停一批不达标企业。加大标准化厂房建设和推广使用力度，落实减税降费政策，实施好铁路货运“一口价”下浮、天然气“保量稳价”、直购电等政策，推动甘河工业园区电力局域网建设，全方位降低企业成本。深化“放管服”改革，试行简易审批事项网上预审，年内网上审批率达到90%。进一步优化审批流程，探索推广创业事项“一窗通办”，努力实现“最多跑一次”。开展优化营商环境专项整治活动，坚持“两个毫不动摇”，落实非公经济15条政策措施，切实解决制约非公企业发展的困难问题，维护非公企业合法权益，健全非公经济发展联席会议制度，构建亲清新型政商关系。深化财税金融改革，发展绿色金融，出台金融支持绿色发展实施方案，支持节能环保、清洁生产、清洁能源等绿色产业发展。严禁地方政府违规举债融资，坚决遏制隐性债务增长，守住不发生系统性金融风险这条底线。健全完善以管资本为主的国有资产监管体制，整合经营性国有资产，逐步实行集中统一监管，引导资本向战略性新兴产业集中。支持城投、湟投等企业发行债券，提高直接融资特别是股权融资比重，降低融资成本和杠杆率。

全面扩大对外开放。力争年内完成西宁综合保税区报批并启动建设，2020年封关运营。青海丝绸之路国际物流城年内建成14万平方米仓储设施，全力打造区域性物资分拨和配送中心。南川片区会展产业集群完成青海丝路国际会展中心前期工作。深化国际产能合作，大力培植外向型优势产业集群，加快建设新能源、新材料等外向型产业基地，推动锂电、光伏、生物医药、绿色食品等优势产业“走出去”，力争全市进出口总额增长8%以上。加强与“一带一路”沿线国家和地区的经贸合作，建立完善国际友城合作机制，办好“第三届凉爽城市国际论坛”和环保大会。

三、进一步加强政府自身建设

各位代表，新时代、新目标，政府要有新气象，更要有新作为。我们将切实做到信念过硬、政治过硬、责任过硬、能力过硬、作风过硬，努力建设人民满意的服务型政府。

一是强化政治建设。政府是党领导下的人民政府，必须旗帜鲜明讲政治，牢固树立“四个意识”，坚决维护以习近平同志为核心的党中央权威和集中统一领导。坚持不懈用习近平新时代中国特色社会主义思想武装头脑、指导实践、推动工作。发挥党组在政府工作中的领导核心作用，忠实践行“两个绝对”，坚决贯彻党中央路线方针政策，当好省市委决策的忠实执行者。

二是强化法治建设。把依法行政作为加强政府自身建设的关键，依照宪法法律行使职权、履行职责，开展“法治政府建设提升行动”，提高运用法治思维和方式深化改革、推动发展、化解矛盾、维护稳定能力。加强政务公开，强化审计工作，推进政务阳光透明。认真执行市人大及其常委会各项决议决定，积极支持人民政协履行职能，认真办理人大议案建议和政协提案，自觉接受人大依法监督、政协民主监督。

三是强化廉政建设。落实全面从严治党主体责任，切实履行“一岗双责”，营造风清气正的政治生态，强化政府系统党风廉政建设。严格执行中央八项规定实施细则精神及省市贯彻措施，深入整治“四风”。强化干部廉政教育和管理，坚守廉政底线，不越纪律红线。严厉惩治群众身边的微腐败，切实维护群众利益。

四是强化作风建设。强化政治理论和业务学习，全面增强“八种本领”，注重提高行政质量，持续提升行政效率。大兴调查研究之风，推动工作流程再造，严格执行“710”和重点工作清单式管理制度，始终保持永不懈怠、永远奋斗的精神状态，以“钉钉子”精神推进各项工作落细落地，强化政府工作执行力。

各位代表，风正潮涌，自当扬帆破浪；任重道远，更需快马加鞭。让我们更加紧密地团结在以习近平同志为核心的党中央周围，在省委、省政府和市委的坚强领导下，团结依靠全市各族人民，不忘初心、牢记使命，抢抓机遇、奋勇争先，在新的历史起点上，拥抱新时代，开启新征程，续写新辉煌，为打造绿色发展样板城市、建设幸福西宁而努力奋斗！
